# Ultrasound‐Assisted Recovery of Chlorogenic Acid From Artichoke (*Cynara scolymus* L.) Leaves: Process Optimization by Response Surface Methodology and Antioxidant Evaluation of the Optimized Extract

**DOI:** 10.1155/ijfo/4423472

**Published:** 2026-10-21

**Authors:** Latifa Thabti, Hamza Ben Abdallah, Rim Hamdi, Amira Horchani, Aimen Abbassi, Amine Trabelsi

**Affiliations:** ^1^ Research Unit for the Pharmaceutical Valorization of Medicinal Plants Faculty of Pharmacy University of Monastir Monastir Tunisia um.rnu.tn

**Keywords:** artichoke leaf (*Cynara scolymus* L.), chlorogenic acid, optimization, response surface methodology, ultrasound-assisted extraction

## Abstract

Artichoke (*Cynara scolymus L.*) is recognized for its numerous health benefits, attributed mainly to its phenolic compounds, such as cynarin and chlorogenic acid, which have antimicrobial, anti‐inflammatory, and antioxidant activities. This study is aimed at enhancing the value of the nonedible parts of the plant by optimizing a method for extracting chlorogenic acid from artichoke leaves. The extraction process of bioactive compound was optimized using the response surface methodology coupled with a Box–Behnken design. A total of 27 experiments were performed, varying three operating parameters: ethanol concentration (from 20% to 70%), sonication time (30 and 45 min), and solid/solvent ratio (1/10 and 1/100 g/mL). The yield of chlorogenic acid was quantified by high‐performance liquid chromatography. The results revealed that the optimized conditions were a solid/solvent ratio of 1/10 g/mL, an ethanol concentration of 56.09%, and a sonication duration of 45 min. The maximum yield of chlorogenic acid was determined to be 14.93%. Under the above‐mentioned conditions, the antioxidant activity was evaluated in vitro using two methods: DPPH and ABTS radical‐scavenging activity. The inhibition concentration 50 (IC50) obtained were 0.230 mg/L for DPPH and 0.237 mg/L for ABTS.

## 1. Introduction

Artichokes have been used as food and medicinal remedies with ancient Egyptian, Greek, and Roman cultures [1]. As archeological remains, the mosaics from the Roman Empire, which are included in the Bardo National Museum in Tunis, confirm that this plant has been in Tunisia since the first century [2].

*Artichoke, scientifically known as Cynara scolymus L.*, belongs to the Asteraceae family. It is a large herbaceous perennial with rosette leaves that are strongly veined and not spiny. These leaves can grow up to approximately 70 × 30 cm and have a deeply indented blade in the upper part. The leaf segments are toothed along the edges but without spines [3]. Artichoke is an important element of the Mediterranean diet and a source of minerals, vitamins, and dietary components. Not only does it contain a high concentration of inulin but also a huge quantity of natural antioxidants, including vitamin C, hydroxycinnamic acids, flavonoids, and anthocyanins [4].

Many experimental studies have talked about the different leaves of *Cynara scolymus*, which prove that it has many pharmacological properties, including hepatoprotective, anti‐inflammatory, antioxidant, and antiobesity effects. All of this gives the probability of using it in therapeutic applications [5–7].

Among these compounds, chlorogenic acid (CGA) is one of the important phenolic compounds found in artichoke leaves. However, although the edible parts of the plant are widely consumed, large amounts of leaves and other nonedible parts are generated as agricultural and agrifood residues. Despite their richness in bioactive compounds, these residues remain largely underutilized [8, 9]. Their valorization therefore represents an interesting approach for recovering high‐value compounds while reducing agricultural waste.

The challenge is not only to extract chlorogenic acid from artichoke leaves but also to identify the extraction conditions that maximize its recovery. Therefore, this study is aimed at evaluating the effect of ethanol concentration, sonication time, and solid/solvent ratio on the CGA yield using response surface methodology (RSM) to optimize these conditions for maximizing the desired responses.

RSM is a statistical approach to evaluate the effect of one variable, which can help build a mathematical relationship between controlled variables and observed outcomes. The most popular design is Box–Behnken; it involves conducting experiments to model interactions between variables. This model uses a second‐degree polynomial equation that includes the measured response, coded variables, and coefficients, representing the influence of each factor and their interactions [10].

This approach provides a sustainable way to valorize artichoke residues. As the extract from the inedible parts of artichoke is rich in CGA, it could be used as an active ingredient in high‐value products, such as dietary supplements and cosmetics. This valorization could contribute to reducing agricultural waste and promoting a circular economy.

## 2. Materials and Methods

### 2.1. Plant Material

Artichoke leaves were collected from the Potato and Artichoke Technical Center located in the “Jdeideh” delegation of Manouba, in northern Tunisia, in January 2023. Two different varieties were collected: the Oranais and the Hyeres. The artichoke leaves were shade‐dried in a well‐ventilated room at ambient temperature (20°C–25°C) without exposure to direct sunlight over a period of 2 weeks. Shade‐drying was selected specifically to minimize the degradation or evaporation of volatile and bioactive components.

### 2.2. Chemicals and Reagents

2,2‐Diphenyl‐1‐picrylhydrazyl (DPPH), 2,2′‐azinobis(3‐ethylbenzothiazoline‐6‐sulphonic acid) diammonium salt (ABTS), and CGA were purchased from Sigma‐Aldrich (St. Louis, Missouri). Ethanol was purchased from Polychem. Methanol and acetonitrile were obtained from Loba Chemie Pvt. Ltd. (Mumbai, Maharashtra, India).

### 2.3. Selection of Variety

Before starting in the phase of optimization, the CGA yield of each batch from both varieties was assessed using the *European Pharmacopoeia* (11th edition) method [11]. Exactly, 0.5 g of leaf powder was added to 50 mL of methanol and heated under a reflux condenser on a water bath at 70°C for 1 h.

Then, we analyze the extracts that we get by high‐performance liquid chromatography (HPLC) as follows (see section determination of CGA yield). The extraction was performed three times; in each time, the mean CGA yield was calculated and compared using appropriate statistical tests. As last step in this phase, we choose the variety that contains the highest CGA′s level.

### 2.4. Ultrasonic Extraction Procedure

The powdered artichoke leaf material was extracted using water–ethanol mixture ranging in concentration from 20% to 70% (Factor B), at a solid/solvent ratio of 1/10–1/100 g/mL (Factor C), into an ultrasonic bath at a fixed frequency of 30 kHz for a period of 30–45 min (Factor A). The obtained extract was filtered, dried under rotary evaporator, and stored at −20°C until further analysis. The ultrasonic extraction was performed using an Elmasonic S 30 ultrasonic water bath (Elma Schmidbauer GmbH, Germany). The temperature was monitored with a digital thermometer, and sonication time was controlled with a chronometer.

### 2.5. Determination of CGA Yield

The CGA content of the drug was determined through HPLC, in accordance with the artichoke leaf monograph from the *European Pharmacopoeia* [11]. Separation was performed using a Shimadzu HPLC system, which includes an autosampler, pump, column furnace, SPD‐20A diode array detector, and a base‐deactivated octadecylsilyl silica gel column measuring 4.6 × 250 mm. The mobile phases were prepared as follows:

• Mobile Phase A: phosphoric acid and water (0.5:99.5 v/v).
• Mobile Phase B: phosphoric acid and acetonitrile (0.5:99.5/v).

The injection volume was set at 25 *μ*L for all runs, and column temperature was set at 40°C. Detection occurred at a wavelength of 330 nm, with a flow rate of 1 mL/min and analysis duration of 40 min. CGA standard was dissolved in methanol at 100 mg/L. Five milliliters of this solution were transferred in a volumetric flask, added with 5 mL of methanol and diluted to 20 mL with water to yield the standard solution. CGA within artichoke leaf extracts was identified based on the standard retention time (RT). The percentage content of CGA is calculated using the following formula:

Y=A12∗m∗pA21∗m∗100,

where

• *Y* is the CGA content;
• A1 is the area of the peak because of CGA in the chromatogram obtained with the solution under examination;
• A2 is the area of the peak because of CGA in the chromatogram obtained with the control solution;
• m1 is the mass of the dried leaf powder sample in grams;
• m2 is the gram mass of CGA used to prepare the control solution, in grams; and
• p is the purity percentage of the CGA reference standard (98%).

### 2.6. Experimental Design

A Box–Behnken experimental design was used to investigate the response variable *Y*, which is considered CGA yield. The independent factors considered were A (sonication time), B (solvent concentration), and C (solvent/solid ratio). The levels of independant variables are presented in Table 1. To estimate the experimental error, we carried out two experiments for each experimental point and three experiments for the experimental point in the center (0, 0, and 0). In all, the experimental design included a total of 27 experiments to obtain 27 dry extracts. All experiments were carried out in random order to reduce the effects of uncontrolled variables which could introduce systematic errors into the measured responses.

**Table 1:** Levels of independent variables for Box–Behnken design.

Independent variables	Codes	Variable levels		
		−1	0	+1
A: sonication time (min)	X1	30	37.5	45
B: ethanol concentration (%)	X2	20	45	70
C: solid/solvent ratio (g/mL)	X3	1/10	1/55	1/100

### 2.7. Model Validation

The extraction was conducted under the identified optimal conditions to validate the precision of the predictive models. The CGA yield of the as‐prepared extract was quantified and compared with the model predicted values of responses.

### 2.8. Estimation of Antioxidant Activities

The antioxidant activity of the optimized extract was assessed by testing the scavenging of ABTS+ and DPPH free radicals.

### 2.9. 2,2‐Diphenyl‐1‐picrylhydrazyl Radical Scavenging Assay

The DPPH radical scavenging activity was evaluated using a modified version of Miliauskas et al.′s protocol [12]. Extracts and ascorbic acid were tested at different concentrations, and absorbance was measured at 517 nm after a 30‐min incubation in the dark at room temperature. Solvent without extract served as negative control, whereas vitamin C was used as positive control. Antioxidant activity was expressed as percentage inhibition (PI) and calculated as follows: PI(%)=(A0−As)/A0×100

where A0 is the negative control absorbance and As is the sample absorbance [13, 14].

### 2.10. 2,2′‐Azinobis (3‐ethylbenzothiazoline‐6‐sulphonic Scavenging Assay

The antioxidant activity of a plant extract was evaluated using the ABTSradical inhibition assay. After preparing the ABTS solution, the extract and ascorbic acid (as reference) were tested at various concentrations. After waiting 7 min incubation in darkness, the absorbance was measured at 734 nm. Antioxidant activity was expressed as PI, with three replicates per concentration [15].

The IC50 value for DPPH and ABTS assays, which represents the concentration needed to neutralize 50% of free radicals, for the various extracts, was determined graphically by plotting PI against extract concentrations using GraphPad Prism 8 software, and this was compared with the IC50 value of vitamin C (positive control).

### 2.11. Statistical Analysis

We use Design Expert 13 software to analyze the experimental data. An analysis of variance (ANOVA) was used to evaluate the statistical significance of the model, with a significance level established at 5%. The model′s fit was determined by calculating the coefficient of determination (*R* ^2^). The relationship between response variables and independent variables (factors) was approved response by surface analysis. For comparative assessments between the two varieties, the experiments were performed in triplicate, and differences between the two varieties were assessed using the nonparametric Mann–Whitney *U* test. A *p* value < 0.05 was considered statistically significant.

## 3. Results and Discussion

### 3.1. Selection of Variety

The CGA yield in artichoke leaves was analyzed via HPLC across the two varieties. The results are summarized in Table 2. A comparison of the experimental values from the two samples revealed no significant difference (*p* > 0.05). We selected the Oranais variety to enhance the extraction efficiency of CGA.

**Table 2:** Comparison between two varieties of artichoke.

Variety	Hyeres variety	Oranais variety
	Mean (sd)^a^	Mean (sd)^a^
Chlorogenic acid (CGA) content (%)	0.49 (0.135)	0.87 (0.34)

*Note:* ^a^Standard deviation.

### 3.2. Model Analysis and Adequacy

The responses of CGA yield obtained from all are shown in Table 3. Experimental data were fitted to a quadratic model. Regression analysis (Table 4) was conducted to estimate the coefficients of the models′ shown terms. The resulting second‐order polynomial equation (Equation 1) was given as follows:

Y=0.59670.935010.415625.9530.032512+ X+ X− X− XX−0.4175130.216323122232 XX− XX+1.79X−0.674X+5.1X,

**Table 3:** Experimental design and observed response.

No. of run	Coded variable			Observed responses
	A: time sonication	B: solvent concentration	C: solid/solvent ratio	Chlorogenic acid yield
	min	%	g/mL	%
3	0 (37.5)	−1 (20)	1 (1/100)	0.02
24	0 (37.5)	−1 (20)	1 (1/100)	0.03
1	−1 (30)	0 (45)	1 (1/100)	0.08
6	−1 (30)	−1 (20)	0 (1/55)	0.1
10	1 (45)	0 (45)	1 (1/100)	0.11
20	−1 (30)	0 (45)	1 (1/100)	0.11
14	−1 (30)	−1 (20)	0 (1/55)	0.13
21	1 (45)	0 (45)	1 (1/100)	0.13
2	−1 (30)	1 (70)	0 (1/55)	0.29
8	0 (37.5)	0 (45)	0 (1/55)	0.5
16	−1 (30)	1 (70)	0 (1/55)	0.58
26	0 (37.5)	0 (45)	0 (1/55)	0.59
9	0 (37.5)	1 (70)	1 (1/100)	0.7
27	0 (37.5)	0 (45)	0 (1/55)	0.7
25	0 (37.5)	1 (70)	1 (1/100)	1.3
7	1 (45)	−1 (20)	0 (1/55)	2.65
17	1 (45)	1 (70)	0 (1/55)	2.7
15	1 (45)	−1 (20)	0 (1/55)	3.47
12	1 (45)	1 (70)	0 (1/55)	3.8
23	0 (37.5)	1 (70)	−1 (1/10)	7.22
22	0 (37.5)	−1 (20)	−1 (1/10)	8.54
4	0 (37.5)	−1 (20)	−1 (1/10)	8.7
13	−1 (30)	0 (45)	−1 (1/10)	10.7
19	1 (45)	0 (45)	−1 (1/10)	13.05
5	0 (37.5)	1 (70)	−1 (1/10)	13.7
18	−1 (30)	0 (45)	−1 (1/10)	17.36
11	1 (45)	0 (45)	−1 (1/10)	18.4

**Table 4:** Estimated regression coefficients for CGA yield.

Source	Sum of squares	Df	Mean square	*F*‐value	*p*	
Model	765.20	9	85.02	14.89	< 0.001	Significant
A: time sonication	13.99	1	13.99	2.45	0.1360	
B: solvent concentration	2.76	1	2.76	0.4841	0.4960	
C: solid/solvent ratio	566.32	1	566.32	99.18	< 01	
AB	0.0085	1	0.0085	0.0015	0.9698	
AC	1.39	1	1.39	0.2442	0.6275	
BC	0.3741	1	0.3741	0.0655	0.8010	
A^2^	18.14	1	18.14	3.18	0.0925	
B^2^	2.57	1	2.57	0.4492	0.5117	
C^2^	147.08	1	147.08	25.76	< 01	
Residual	97.07	17	5.71			
Lack of fit	38.38	3	12.79	3.05	0.0635	Not significant
Pure error	58.68	14	4.19			
Cor total	862.27	26				

where **Y** is the CGA yield and **X**1, **X**2, and **X**3 are the levels of the independent variables time, ethanol concentration, and solid/liquid ratio, respectively, expressed as encoded values.

ANOVA was used to assess model′s adequacy (Table 4) showing a highly significant regression (*p* < 0.001) as well as a nonsignificant lack of fit (*p* > 0.05). Additionally, the coefficient of determination (*R* ^2^) for the model was 0.874, whereas the predicted *R* ^2^ reached 0.711, demonstrating a reasonable concordance with the adjusted *R* ^2^ of 0.827. Taken together, these statistical indicators strongly support the validity and robustness of the proposed model.

As shown in Table 4, the linear coefficient (C) and the quadratic coefficient (C^2^) are statistically significant (*p* < 0.05). The solvent/solid ratio factor exhibits a significant effect, indicating that variations in this parameter are associated with measurable changes in the response. It is therefore a key factor influencing the outcome, and its variation should be carefully controlled and optimized. However, the other two factors, ethanol concentration and sonication time, do not have a statistically significant effect on the CGA content. This suggests that changes in these factors do not lead to substantial variations in the measured response.

### 3.3. Response Surfaces Plots

Two of the variables are varied within the experimental range, whereas the third variable is held constant at zero level. Figures 1–3 represent response surfaces and contour plots of interaction between CGA yield sonification time and ethanol concentration. Figure 2 shows that the maximum CGA yield is achieved at low solid/solvent ratios and longer extraction times. This could be because of the enhancement of mass transfer upon reduction of solid/solvent ratio, allowing solutes like CGA to be transferred smoothly from plant to liquid solvent [16].

**Figure 1:**
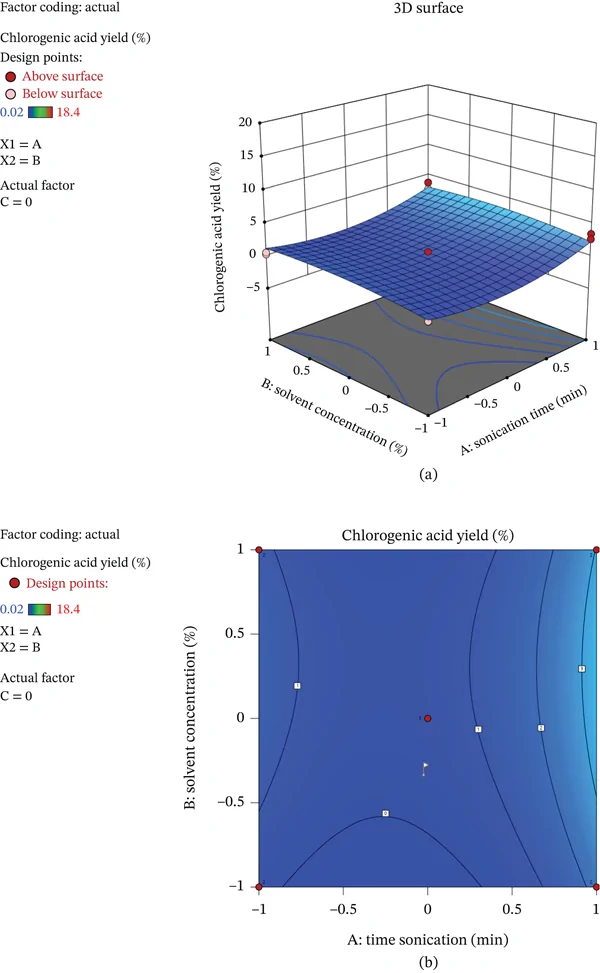
Response surface graph for chlorogenic acid yield, which is derived from artichoke leaves as a function of sonication time ratio to solvent concentration. (a) 3D surface. (b) Contour curves.

**Figure 2:**
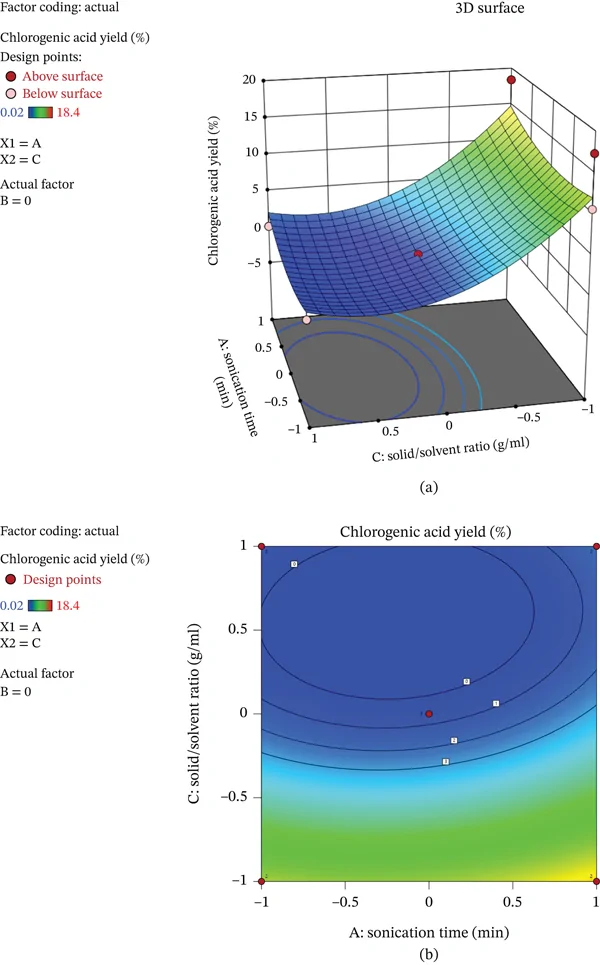
Response surface graph for chlorogenic acid yield, which is derived from artichoke leaves as a function of sonication time ratio to solid/solvent ratio. (a) 3D surface. (b) Contour curves.

**Figure 3:**
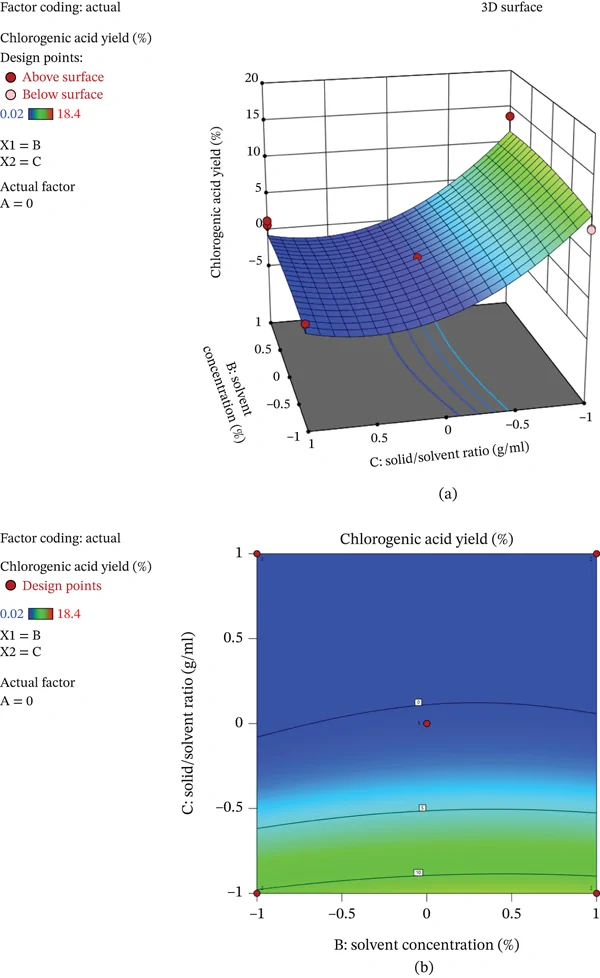
Response surface spot for CGA yield, which is derived from artichoke leaves, as a function of solvent concentration ratio to solid/solvent ratio. (a) 3D surface. (b) Contour curves.

Figure 3 shows that maximum CGA yield occurs at low solvent‐to‐solid ratio values with a concentration of about 50% ethanol. The CGA yield displays no significant change with changing extraction time and ethanol content. A nearly flat response surface plot (i.e., none of these variables significantly influences the response as measured) manifests this. This corroborates earlier ANOVA analysis (Table 4). Attention should then be focused on solid/solvent factors, which have a greater impact on the response, thus streamlining the optimization process.

Solid/solvent ratio variables must thus be the area of concern because they exert a more intense influence on the response, thereby making the process easier to optimize. Some studies have established that the most important parameters affecting the extraction of antioxidants from plant material include solvent concentration, extraction time, and solid/solvent ratio. Solvent concentration influences polarity: With reduced ethanol concentration in an aqueous solution, solvent polarity is increased. Through the modification of the solvent′s polarity, we control the solubility of desired solutes—in this instance, CGA—and, therefore, their extraction yield [17].

Incubation time is dependent upon the time required for maceration of artichoke leaf particles and migration of the phenolic compounds from the particles to the solvent. Saha et al. [18] have reported that food phenolic compounds are better extracted with an increase in extraction time, ranging from 30 to 150 min.

Increasing the solvent volume relative to the amount of plant material can enhance antioxidant extraction by improving the solubilization and mass transfer of bioactive compounds. But a very high ratio will not be ideal, as it may result in the dilution of the resultant compounds, with increased cost and operation complexity without achieving a significant improvement in the yield [19].

### 3.4. Model Validation

The model was validated and verified by both experimental and statistical confirmation, demonstrating its effectiveness in well describing and predicting the phenomenon being studied. As it was found under the proposed model, the best conditions for achieving maximum CGA yield were 56.09% ethanol concentration, 45 min sonication time, and 1/10 g/mL solvent‐to‐plant material ratio. These conditions yielded the highest CGA yield, reaching 14.93%. After statistical validation, the initially hypothesized model was experimentally verified to further evaluate its predictive efficiency. The best test point in this respect was chosen, and the resulting output is shown in Table 5.

**Table 5:** Predicted and measured responses values of the optimized extract.

Solid/solvent ratio (g/mL)	Optimum conditions		Chlorogenic acid yield	
	Ethanol concentration (%)	Sonication time (min)	Predicted values mean (sd^a^)	Experimental values mean (sd^a^)
1/10	56.09	45	14.92% (2.39)	12.45% (0.5)

*Note:* ^a^Standard deviation.

The model predicted a CGA yield of 14.93%, while the experimental value was 12.45%. The experimental value fell within the 95% confidence interval of the predicted response, indicating satisfactory agreement between the predicted and experimental values.

### 3.5. Optimization of Parameters for Extraction

Only a few studies have been conducted to optimize the extraction process of CGA from artichoke leaves. Other factors such as sonication frequency and extraction temperature have also been researched. According to a study by Atıcı and Bulduk [20], the highest yield of CGA (10.05 ppm) was obtained from artichoke leaves under the following optimal conditions: ultrasonic power of 47.65%, an extraction time of 35.18 min, and an extraction temperature of 53.40°C.

On the other hand, Solano et al. [21] optimized ultrasound‐assisted extraction of polyphenols from artichoke bracts and found that the best conditions were 97% ultrasound amplitude, a 53% ethanol concentration, and a 9.7‐min extraction time. Although the plant material used (bracts) differed from our study (leaves), the ethanol concentration is similar (52.6%); their extraction time is also considerably shorter than ours (45 min).

Differences between study results are explained by several factors related to the plant material itself, such as the nature of the matrix, the species used, and the granulometry of the powder, as well as operating conditions, including the extraction technique, solvent type, and ultrasound frequency. In addition, the operating conditions such as the extraction technique, the type of solvent used, and the frequency of ultrasound [22,23]. In another study, Melini et al. [24] optimized the ultrasound‐assisted extraction of phenolic compounds from artichoke heads using RSM. The optimal conditions were as follows: an extraction time of 20 min, a temperature of 60°C, and solvent to sample ratio of 50 mL/g. HPLC analysis showed that caffeoylquinic acid derivatives, particularly cynarin and 1,5‐O‐dicaffeoylquinic acid, were the predominant compounds.

### 3.6. Antioxidant Activities

In this study, the in vitro antioxidant activity of the samples was evaluated using the DPPH and ABTS assays. The results of the DPPH test (Figure 4) show that the antifree radical activity of the optimized extract is lower than that of vitamin C and gradually increased with increasing concentration. At a concentration of 1 mg/mL, the percent inhibition rate reached 86.64%. The calculated IC_s_50 (mg/mL) for extract and vitamin C were 0.294 (0.068) and 0.082 (0.0005), respectively. Several studies have shown that the antioxidant capacity of artichoke leaves is primarily due to their high content of phenolic compounds. Wang et al. [25] found that methanolic extracts of artichoke leaves showed a strong antioxidant capacity, with caffeoylquinic acid derivatives, including cynarin and 1,5‐O‐dicaffeoylquinic acid, as the main compounds. In another study by Pandino et al. [26] showed that different varieties of artichoke leaves exhibited DPPH inhibition values ranging from 70% to 90%, with the “Campagnano” variety displaying particularly high antioxidant activity, mainly attributed to CGA and luteolin‐7‐O‐glucoside.

**Figure 4:**
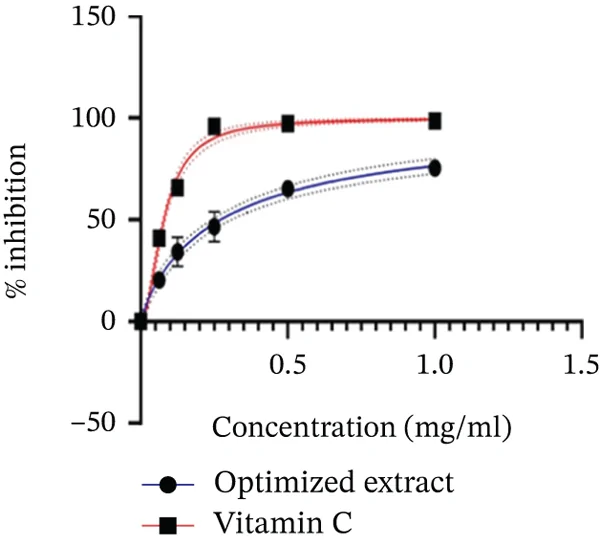
DPPH scavenging activity of optimized extract versus vitamin C.

These findings highlight the potential of artichoke leaves as a natural source of antioxidants. The antifree radical potential of artichoke leave extracts was also evaluated against a second type of radical, the ABTS+ cation. Figure 5 shows that, similarly to DPPH results, the percentage of ABTS inhibition increases with the increase of extract concentration. The IC_s_50 (mg/mL) of the optimized extract and vitamin C were 0.221 (0.006) and 0.085 (0.068), respectively.

**Figure 5:**
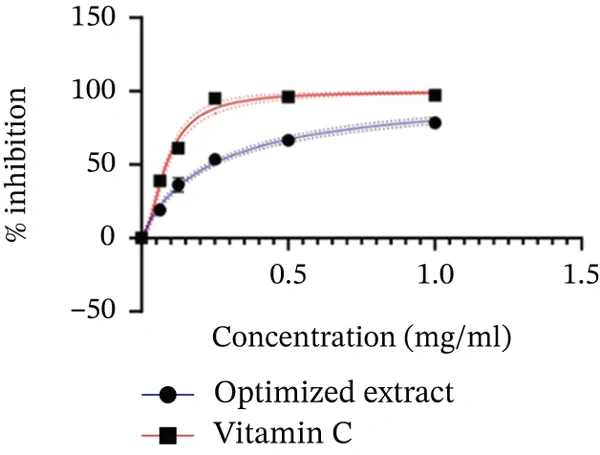
ABTS scavenging activity of optimized extract versus vitamin C.

## 4. Conclusion

A Box–Behnken design was used to optimize ultrasound‐assisted extraction of CGA from artichoke leaves through 27 experiments. The optimal conditions were identified as a solid‐to‐solvent ratio of 1 g/10 mL, 56.09% ethanol, and a 45‐min extraction time. The resulting CGA‐rich extract shows potential for further investigation of its biological activities. However, some limitations should be acknowledged. The study considered only three variables, excluding other important factors such as temperature and ultrasonic power, and the selected ranges may not cover the true optimum. Moreover, the Box–Behnken design is limited to a quadratic model and may not capture complex interactions. In addition, plant‐related factors such as particle size, drying conditions, and variability in artichoke origin were not considered.

## Author Contributions

Latifa Thabti: Material preparation, data curation, formal analysis and investigation, and writing—original draft preparation. Hamza Ben Abdallah: Conceptualization, methodology, formal analysis and investigation, and writing—review and editing. Rim Hamdi: Material preparation, investigation, and data curation. Amira Horchani: Conceptualization, methodology, supervision, and editing. Aimen Abbassi: Supervision and editing. Amine Trabelsi: Conceptualization, methodology, investigation, and supervision.

## Funding

No funding was received for this manuscript.

## Conflicts of Interest

The authors declare no conflicts of interest.

## Data Availability

The data that support the findings of this study are available from the corresponding author upon reasonable request.

## References

[1] De Falco B. , Incerti G. , Amato M. , and Lanzotti V. , Artichoke: Botanical, Agronomical, Phytochemical, and Pharmacological Overview, Phytochemistry Reviews. (2015) 14, no. 6, 993–1018, 10.1007/s11101-015-9428-y.

[2] Abdelfattah S. , Bianco V. V. , Calabrese N. , and Dridi B. , Guide de Bonnes Pratiques de la Culture D′artichaut Conduite en Mode Raisonnée, 2010, Ministère de l′Agriculture, des Ressources Hydrauliques et de la Pêche.

[3] Sharma P. , Verma P. K. , Pankaj N. K. , and Agarwal S. , The Phytochemical Ingredients and Therapeutic Potential of *Cynara scolymus* L., Pharmaceutical and Biomedical Research. (2022) 7, no. 3, 141–160, 10.18502/pbr.v7i3.7696.

[4] Rejeb I. B. , Dhen N. , Gargouri M. , and Boulila A. , Chemical Composition, Antioxidant Potential and Enzymes Inhibitory Properties of Globe Artichoke By-Products, Chemistry & Biodiversity. (2020) 17, no. 9, e2000073, 10.1002/cbdv.202000073, 32628807. 32628807

[5] Hayata M. , Watanabe N. , Kamio N. , Tamura M. , Nodomi K. , Tanaka K. , Iddamalgoda A. , Tsuda H. , Ogata Y. , Sato S. , Ueda K. , and Imai K. , Cynaropicrin From *Cynara scolymus* L. Suppresses *Porphyromonas gingivalis* LPS-Induced Production of Inflammatory Cytokines in Human Gingival Fibroblasts and RANKL-Induced Osteoclast Differentiation in RAW264.7 Cells, Natural Medicines. (2019) 73, no. 1, 114–123, 10.1007/s11418-018-1250-6, 30218207. 30218207

[6] Miraj S. and Kiani S. , *Cynara scolymus* L.: A Review Study of Its Phytochemical and Pharmacological Properties, Der Pharmacia Lettre. (2016) 8, no. 19, 348–352.

[7] Saénz Rodriguez M. T. , García Giménez D. , and de la Puerta Vázquez R. , Choleretic Activity and Biliary Elimination of Lipids and Bile Acids Induced by an Artichoke Leaf Extract in Rats, Phytomedicine: International Journal of Phytotherapy and Phytopharmacology. (2002) 9, no. 8, 687–693, 10.1078/094471102321621278, 12587687. 12587687

[8] Lavecchia R. , Maffei G. , Paccassoni F. , Piga L. , and Zuorro A. , Artichoke Waste as a Source of Phenolic Antioxidants and Bioenergy, Waste and Biomass Valorization. (2019) 10, no. 10, 2975–2984, 10.1007/s12649-018-0305-y.

[9] Rudić S. , Dimitrijević-Branković S. , Dimitrijević S. , and Milić M. , Valorization of Unexploited Artichoke Leaves Dust for Obtaining of Extracts Rich in Natural Antioxidants, Separation and Purification Technology. (2021) 256, 117714, 10.1016/j.seppur.2020.117714.

[10] Myers R. H. , Montgomery D. C. , and Anderson-Cook C. M. , Response Surface Methodology: Process and Product Optimization Using Designed Experiments, 2016, 4th edition, Wiley, https://www.wiley.com/en-us/Response+Surface+Methodology%253A+Process+and+Product+Optimization+Using+Designed+Experiments%252C+4th+Edition-p-9781118916018.

[11] European Directorate for the Quality of Medicines & HealthCare (EDQM) , European Pharmacopoeia, 2023, 11th edition, Council of Europe.

[12] Miliauskas G. , Venskutonis R. P. , and van Beek T. A. , Screening of Radical Scavenging Activity of Some Medicinal and Aromatic Plant Extracts, Food Chemistry. (2004) 85, no. 2, 231–237, 10.1016/j.foodchem.2003.05.007.

[13] Biel W. , Witkowicz R. , Piątkowska E. , and Podsiadło C. , Proximate Composition, Minerals and Antioxidant Activity of Artichoke Leaf Extracts, Biological Trace Element Research. (2020) 194, no. 2, 589–595, 10.1007/s12011-019-01806-3, 31286388. PMC7015959 31286388

[14] Dieng S. I. , Mbacké A. D. , Fall K. D.-B. , Sarr A. , Sene M. , Sene M. , Mbaye A. , Diatta W. , and Bassene E. , Evaluation de l′activité Antioxydante des Extraits Hydro-Ethanoliques des Feuilles et Écorces de *Piliostigma thonningii* Schumach, International Journal of Biological and Chemical Sciences. (2017) 11, no. 2, 10.4314/ijbcs.v11i2.19.

[15] Benali A. , Haremarugira S. , Bitar A. , El Maadoudi E. H. , Rachid K. , and Noutfia Y. , Composition en Antioxydants et Activité Coagulante des Artichauts Sauvage et Cultivé au Maroc (*Cynara cardunculus* L.), Revue Marocaine des Sciences Agronomiques et Vétérinaires. (2022) 10, no. 2, 200–216.

[16] Oteef M. D. Y. , Comparison of Different Extraction Techniques and Conditions for Optimizing an HPLC-DAD Method for the Routine Determination of the Content of Chlorogenic Acids in Green Coffee Beans, Separations. (2022) 9, no. 12, 10.3390/separations9120396.

[17] Weihs C. and Ickstadt K. , Data Science: The Impact of Statistics, International Journal of Data Science and Analytics. (2018) 6, no. 3, 189–194, 10.1007/s41060-018-0102-5.

[18] Saha J. , Biswas A. , Chhetri A. , and Sarkar P. K. , Response Surface Optimisation of Antioxidant Extraction From Kinema, a Bacillus-Fermented Soybean Food, Food Chemistry. (2011) 129, no. 2, 507–513, 10.1016/j.foodchem.2011.04.108, 30634258. 30634258

[19] Markom M. , Hasan M. , Daud W. R. W. , Singh H. , and Jahim J. , Extraction of Hydrolysable Tannins From *Phyllanthus niruri* Linn.: Effects of Solvents and Extraction Methods, Separation and Purification Technology. (2007) 52, no. 3, 487–496, 10.1016/j.seppur.2006.06.003.

[20] Atıcı Y. and Bulduk İ. , Optimization the Ultrasonic Assisted Extraction of Chlorogenic Acid From the Artichoke (*Cynara Scolymus*) Leaf With Response Surface Methodology and Total Phenolic Contents, Hacettepe Journal of Biology and Chemistry. (2020) 48, no. 2, 147–157, 10.15671/hjbc.511586.

[21] Solano Q. , Miguel J. , Valenzuela A. H. , de la Cruz C. , Silva G. Q. , and Cervantes G. , Optimization of Ultrasound-Assisted Extraction of Polyphenols From Globe Artichoke (*Cynara scolymus* L.) Bracts Residues Using Response Surface Methodology, Acta Scientiarum Polonorum Technologia Alimentaria. (2021) 20, no. 3, 277–290, 10.17306/J.AFS.0937. 34304546

[22] Sinha D. , Mukherjee S. , and Chowdhury S. , Methods of Extraction of Phytochemicals, Dans Extraction Processes of Natural Products, 2022, Wiley, 250–279, 10.4018/978-1-6684-7337-5.ch010.

[23] Stumpf B. , Künne M. , Ma L. , Xu M. , Yan F. , Piepho H.-P. , and Honermeier B. , Optimization of the Extraction Procedure for the Determination of Phenolic Acids and Flavonoids in the Leaves of Globe Artichoke (*Cynara cardunculus* var. *scolymus* L.), Journal of Pharmaceutical and Biomedical Analysis. (2020) 177, 112879, 10.1016/j.jpba.2019.112879, 31542418. 31542418

[24] Melini V. , Melini F. , and Comendador F. J. , Response Surface Methodology as an Experimental Strategy for Ultrasound-Assisted Extraction of Phenolic Compounds From Artichoke Heads, Antioxidants. (2023) 12, no. 7, 10.3390/antiox12071360, 37507900. PMC10376278 37507900

[25] Wang M. , Simon J. E. , Aviles I. F. , He K. , Zheng Q.-Y. , and Tadmor Y. , Analysis of Antioxidative Phenolic Compounds in Artichoke (*Cynara scolymus* L.), Journal of Agricultural and Food Chemistry. (2003) 51, no. 3, 601–608, 10.1021/jf020792b. 12537429

[26] Pandino G. , Lombardo S. , Mauromicale G. , and Williamson G. , Phenolic Acids and Flavonoids in Leaf and Floral Stem of Cultivated and Wild *Cynara cardunculus* L. Genotypes, Food Chemistry. (2011) 126, no. 2, 417–422, 10.1016/j.foodchem.2010.11.001.

